# Cigarette smoke extracts and cadmium induce COX-2 expression through γ-secretase-mediated p38 MAPK activation in C6 astroglia cells

**DOI:** 10.1371/journal.pone.0212749

**Published:** 2019-02-22

**Authors:** Hyun Joung Lim, Jung Hyun Park, Chulman Jo, Keejung Yoon, Young Ho Koh

**Affiliations:** 1 Division of Brain Diseases, Center for Biomedical Sciences, Korea National Institute of Health, Osong-eup, Heungdeok-gu, Cheongju-si, Chungcheongbuk-do, Republic of Korea; 2 College of Biotechnology and Bioengineering, Sungkyunkwan University, Jangan-gu, Suwon-si, Gyeonggi-do, Korea; Institute of Biochemistry and Biotechnology, TAIWAN

## Abstract

Exposure to cigarette smoke has been implicated in the progression of cerebrovascular and neurological disorders like stroke through inflammation and blood-brain barrier disruption. In this study, we investigated the signaling cascade activated by cigarette smoke extracts (CSE) and cadmium (Cd) resulting in the COX-2 induction in C6 rat astroglia cells. CSE or Cd induced Notch1 cleavage and activated p38 MAPK and CREB signaling pathways in C6 astroglia cells. Knockdown of nicastrin using siRNA or γ-secretase inhibitors, DAPT and L-685,486, reduced Notch1 cleavage and phosphorylation of p38 MAPK and CREB, while phosphorylation of ERK and JNK remained unaffected. Additionally, the blockage of γ-secretase activity did not show any effect on the phosphorylation of AKT, another upstream activator of CREB, indicating that γ-secretase-mediated CREB activation occurs via p38 MAPK. γ-secretase inhibitor also inhibited the CSE and Cd-mediated increase in the expression of COX-2. Furthermore, recombinant overexpression of Notch1 intracellular domain resulted in an increase in the expression of COX-2. Notch signaling induced by CSE and Cd induced apoptosis in C6 cells. Our results demonstrate that CSE exposure activated the p38 MAPK and CREB-mediated induction in COX-2 expression in astrocytes via γ-secretase-mediated Notch1 signaling. Our data provides novel insights into the potential mechanism of pro-inflammatory response activated by exposure to cigarette smoke.

## Introduction

Cigarette smoke is reportedly a major risk factor for stroke and vascular diseases [1]. Several environmental pollutants including heavy metals are associated with neurological disorders, such as ischemic stroke and learning disabilities in children [2–4]. Cadmium (Cd), a potent mediator of oxidative stress and inflammation, is an environmental pollutant present in cigarettes and contaminated food. Cd is also one of major components of air particulate matters that associated with acute changes in cardiovascular or respiratory physiology [5]. A few studies report a significant correlation between the increased risk for stroke and Cd or cigarette smoke extract (CSE) exposure [6, 7]. Brain ischemia triggers an inflammatory reaction that contributes to the progression of brain diseases [8].

Astrocytes, a major type of glial cells in the brain, play an important role in stroke and are involved in the regulation of the brain microenvironment and maintenance of the blood-brain barrier [9]. Astrocytes also regulate the cerebral blood flow (CBF) [10]. Production of inflammatory cytokines and toxic mediators by astrocytes has been reported to be associated with stroke pathology [11]. Cyclooxygenase-2 (COX-2), an enzyme mediating the progression of inflammation, plays a critical role in the progression of cerebral ischemic damage. Increased COX-2 expression is observed in rodents and patients with ischemic stroke [12]. Substantial evidence supports the potential effect of cigarette smoke on COX-2 and its downstream metabolites such as prostaglandin E_2_ (PGE_2_) [13] and COX-2 knock-out mice are protected against brain ischemia [14]. Cyclic AMP response element-binding protein (CREB) and activating transcription factor 1 (ATF1) are the major proteins that regulate COX-2 expression [15] and cigarette smoke, in turn, reportedly induces CREB phosphorylation [16]. Since Cd induces COX-2 upregulation via γ-secretase [17], it can be speculated that CREB phosphorylation is involved in γ-secretase-mediated COX-2 upregulation induced by Cd.

Presenilin (PS), also called γ-secretase, is recognized as one of the causes for Alzheimer’s diseases. γ-secretase is a multi-protein complex composed of four proteins, presenilin 1 (PS1) and 2 (PS2), nicastrin, APH-1 (anterior pharynx-defective 1), and PEN-2 (presenilin enhancer 2) [18]. Several proteins, such as amyloid precursor protein (APP), Notch-1, and N-cadherin are substrates for γ-secretase-dependent protein processing [19, 20]. Notch1 is abundantly expressed in neurons and astrocytes and is involved in the mitogen-activated protein kinase (MAPK) signaling cascades to modulate inflammation [21]. Although Notch1 has been shown to worsen stroke outcome through glial cell-mediated inflammatory responses, the molecular mechanisms of γ-secretase dependent association of Notch1 processing with hazardous outcomes of cigarette smoke exposure remain elusive.

Here, we investigated the signal transduction pathways by which Cd or cigarette smoke induce COX-2 expression and apoptosis. Since the COX-2 promoter contains a cyclic AMP response element (CRE), we wondered if p38 MAPK/CREB signaling cascades play a role in mediating the induction of COX-2 via γ-secretase. We show that Cd or cigarette smoke exposure to C6 astrocytes is accompanied by γ-secretase-mediated Notch1 intracellular domain (NICD) production and activation of p38 MAPK signaling and its downstream target CREB, thereby inducing the expression of COX-2. Notch1 signaling induced by cigarette smoke and Cd induces apoptosis in C6 astrocytes. Together, our data suggest that COX-2 overexpression induced by Cd or cigarette smoke in astrocytes involves the activation of p38 MAPK/CREB signaling pathways following γ-secretase-mediated Notch1 cleavage, and regulates apoptosis.

## Materials and methods

### Materials

The γ-secretase inhibitors [N-[N-(3,5-Difluorophenacetyl-Lalanyl)]-S-phenylglycine t-butyl ester (DAPT)], L-685,486, [1,2-bis(o-Aminophenoxy)ethane-N,N,N’,N’-tetraacetic acid tetra(acetoxymethyl) ester (BAPTA-AM)] and SB202190 were purchased from Calbiochem (La Jolla, CA). 3-(4,5-dimethylthiazol-2-yl)-2,5-di-phenyltetrazolium bromide (MTT), Cadmium chloride (CdCl_2_), N-acetylcysteine (NAC) and N-[2-(Cyclohexyloxy)-4-nitrophenyl] methanesulfonamide (NS-398), Bicinchoninic acid protein assay kit were purchased from Sigma (Saint Louis, MO). Anti-cleaved parp (#9545, Asp214/215), anti-cleaved caspase-3 (#9664, Asp175), anti-phospho ERK1/2 (#9101, Thr202/Tyr204), anti-ERK1/2 (#9102), anti-stress-activated protein kinase (SAPK)/JNK (#9252), anti-phospho SAPK/JNK (#9251, Thr183/Tyr185), anti-p38 MAPK (#9212) and anti-phospho p38 MAPK (#9211, Thr180/Tyr182), anti-CREB (#9197), anti-phospho CREB (#9198, Ser133), anti-AKT (#9272) and anti-phospho AKT (#9275, Thr308) antibodies were from Cell Signaling Technology (Beverly, MA). Anti-COX-2 (sc-1745) antibody was from Santa Cruz Biotechnology (Santa Cruz, CA). Anti-GFP antibody (A11122) obtained from Molecular Probes (Eugene, OR). Anti-activated notch-1 antibody (ab8925) was from Abcam (Cambridge, MA).

### Cell culture

The C6 rat astroglia cell lines are widely used as an astrocytes-like cell line to study astrocytic function. The C6 cell line used in this study was maintained in Dulbecco’s modified Eagle’s medium (DMEM) supplemented with 10% fetal bovine serum, 100 units/ml penicillin, and 100 μg/ml streptomycin. Cells were maintained at 37°C in humidified atmosphere with 95% air and 5% CO_2_. Cells were pretreated with the inhibitors for 1 h before at 37°C followed by the addition of CdCl_2_ at the designed concentrations and hours. CdCl_2_ was dissolved in distilled water. Vehicle treatment was used as control group.

### Preparation of cigarette smoke extracts

CSE kindly provided by Dr. KwangSoo Lee (KCDC). Cigarette smoke extracts (CSE) were generated by reference cigarettes (3R4F) which were purchased from University of Kentucky (Lexington, KY). 3R4F were conditioned following ISO standard 3402 (at least 48 h at 22±1°C and a relative humidity 60±3%) before smoked. It was smoked on RM20H smoking machine (Borgwaldt KC GmbH, Hamburg, Germany) under ISO standard 3308 (puff volume 35 mL, puff duration 2s, puff interval 60s). Total particulate matter (TPM) was collected on Cambridge filter pads. The final concentration of 10 mg/mL TPM was extracted with dimethyl sulfoxide (DMSO, Sigma) by shaking for 20 min. The solution was then filtered and stored at –80°C. Cells were pretreated with the inhibitors for 1 h before at 37°C followed by the addition of CSE at the designed concentrations and hours. Vehicle treatment was used as control group.

### Cell lysates and western blotting

Cells in plates were washed with phosphate-buffered saline (PBS) and lysed in radioimmunoprecipitation (RIPA) buffer (1% Nonidet P-40, 150 mM NaCl, 50 mM Tris-HCl, pH 7.5, 0.1% sodium dodecyl sulfate, 1 mM phenylmethylsulfonyl fluoride, 0.1 mM Na_3_VO_4_, and 100 μg/ml of leupeptin). The lysates were cleared by centrifugation (15,000 rpm, 5 min, 4°C), and the protein concentrations were determined using the bicinchoninic acid method (Sigma). Equivalent amounts of protein were separated on NuPAGE (4–12%; Invitrogen) gels and transferred to polyvinylidene difluoride membranes. Membranes were blocked in 5% fat-free milk in tris-buffered saline (TBS) with 0.1% tween-20 and incubated with the primary antibody overnight at 4°C. Immune complexes were then detected using the enhanced chemiluminescence system (Amersham, Buckinghamshire, UK).

### Transient transfection

Cells were transiently transfected with the human Notch intracellular domain (NICD) cloned into the IRES-eGFP vector [22]. The Lipofectamine LTX reagent and Opti-MEM medium (Life technology, NY, USA) were used to transfection according to the manufacturer’s instructions. Small interfering RNA (siRNA) specific to nicastrin (L-095053-02) and non-targeting control siRNA (D001810) were purchased from Dharmacon RNA Technology (Lafayette, CO). siRNA was transfected into the respective cell lines at 100 nM (final concentration) using Lipofectamine 2000 (Invitrogen), according to the manufacturer’s instructions.

### Assays of cell viability

Cell viability was determined by the conventional MTT reduction assay [23]. Viable cells convert MTT to insoluble blue formazan crystals by dehydrogenase activity. Cells were incubated in serum-free DMEM for 24 h, followed by treatment with the given concentrations of each compound for the indicated time. Cells were then washed with PBS and treated with MTT solution (2 mg/ml) for 1 h at 37°C. After incubation, cells were dissolved in DMSO. Absorbance was measured with a microplate reader at 570 nm.

### Prostaglandin E_2_ (PGE_2_) quantification

Supernatant from each culture medium was collected by centrifugation (4,000 x g) at 4°C to remove particulate. Supernatant was quantified immediately using prostaglandin E_2_ assay kit (R&D systems, Minneapolis, MN) according to the manufacturer’s protocol. The OD of each well was determined with microplate reader at 450 nm.

### Statistical analysis

Statistical analyses were performed using the Student t-test and ANOVA followed by the Duncan post-hoc test using an SPSS program (SPSS, Inc.). All assays were repeated at least 3 times. P values<0.05 were considered to indicate statistical significance.

## Results

### CSE and Cd increase γ-secretase-mediated NICD level

**Fig 1:**
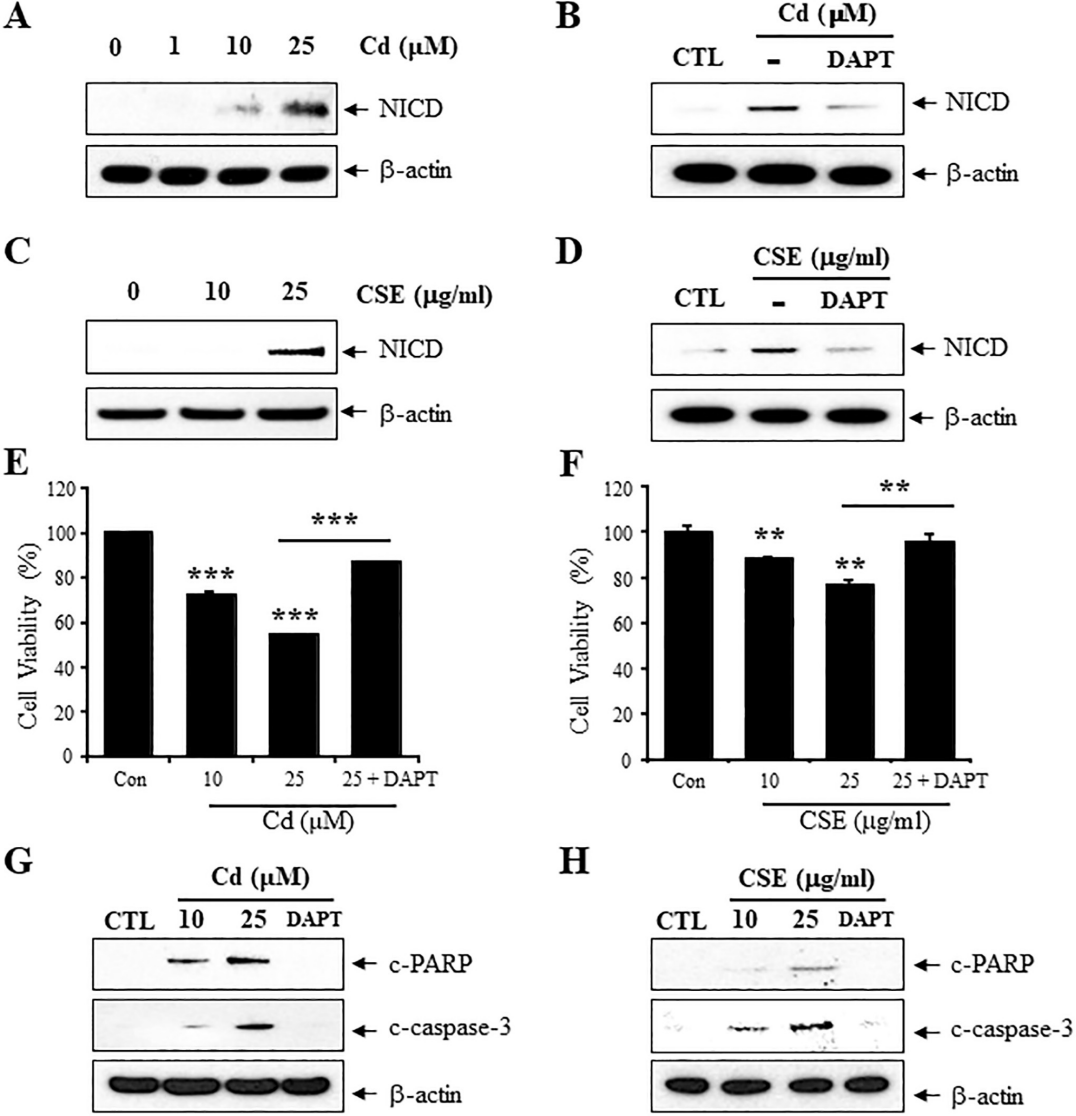
Cigarette smoke extracts and Cd increase γ-secretase-mediated NICD level. (A) C6 cells were treated for 6 h with 0, 1, 10, and 25 μM Cd, lysed in RIPA buffer, and the lysates were probed with antibodies against NICD. (B) C6 cells were pretreated for 1 h with γ-secretase inhibitors (2.5 μM DAPT) followed by exposure to 25 μM Cd for 6 h. (C) C6 cells were exposed 10 and 25 μg/ml CSE, lysed, and the lysates were probed with antibodies against NICD. (D) C6 cells were pretreated for 1 h with γ-secretase inhibitors (2.5 μM DAPT) and then exposed to 25 μg /ml CSE for 6 h. β-actin was used as a loading control for lysates. (E) The effect of Cd on the viability of C6 cells was evaluated by MTT assay. Cells were pretreated for 1 h with γ-secretase inhibitors (2.5 μM DAPT) and then exposed to 25 μM Cd for 6 h. (F) Effect of CSE on the viability of C6 cells was evaluated. Cells were pretreated for 1 h with γ-secretase inhibitors (2.5 μM DAPT) and then exposed 25 μg/ml CSE. Immunoblot analysis of apoptosis-related proteins. (G) Cells were pretreated for 1 h with γ-secretase inhibitors (2.5 μM DAPT) and then exposed to 10, 25 μM Cd for 6 h. (H) Cells were pretreated for 1 h with γ-secretase inhibitors (2.5 μM DAPT) and then exposed to 10, 25 μg/ml CSE for 6 h. The lysates were probed with antibodies against c-caspase-3 (Asp175) and c-PARP (Asp214/215). β-actin was used as a loading control for lysates. Data are shown as the mean ± SD (n = 3), **P<0.01, ***P<0.001, compared to control.

We first observed an increase in the levels of NICD in C6 astrocytes following 6 h treatment with 25 μg/ml CSE or 25 μM Cd (Fig 1A and 1C). The γ-secretase inhibitors (DAPT) blocked the NICD induction by CSE or Cd, suggesting that CSE and Cd-mediated increase in NICD levels is based on activation of γ-secretase in C6 astrocytes (Fig 1B and 1D). MTT based cell viability assay was used to assess the effect of γ-secretase on Cd and CSE-induced cytotoxicity in C6 cells. Exposure to Cd or CSE for 6 h significantly decreased the cell viability in a dose-dependent manner (Fig 1E and 1F). To determine whether apoptosis is responsible for the vulnerability of astrocyte to Cd or CSE induced cell damage, the effect of Cd and CSE on the cellular features of apoptosis was examined with the cleaved caspase-3 (c-caspase-3) and cleaved PARP (c-PARP) (Fig 1G and 1H). The c-caspase-3 (Asp175) and c-PARP (Asp214/215) were increased in Cd or CSE treated cells compared to the control but significantly attenuated with DAPT pretreatment. Consistent with the inhibitory effect on the production of NICD, DAPT treatment partially blocked the CSE and Cd-induced cytotoxicity.

### γ-secretase regulates Cd-induced p38 MAPK phosphorylation

**Fig 2:**
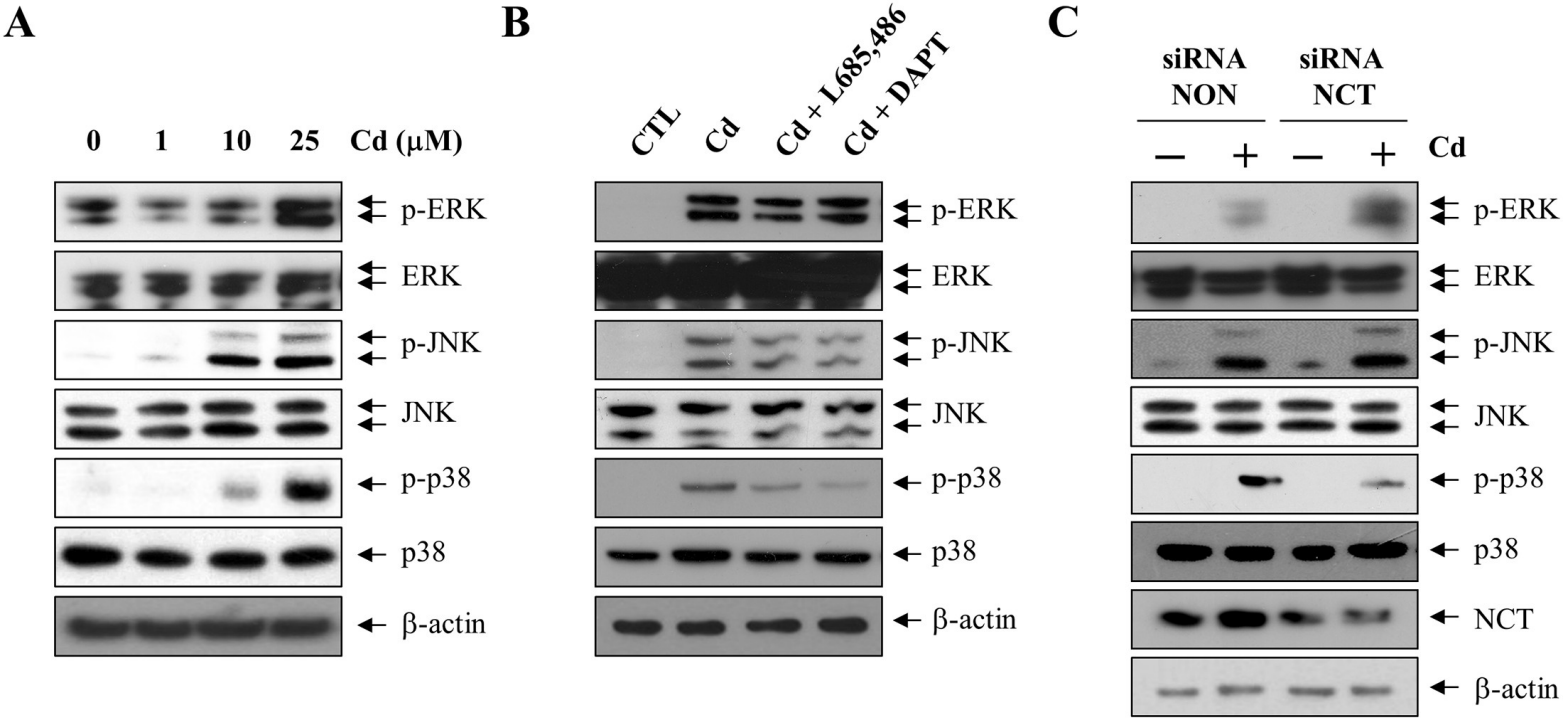
Phosphorylation of p38 MAPK by Cd is mediated by γ-secretase activation. (A) C6 cells were treated for 6 h with 0, 1, 10, and 25 μM Cd, lysed in RIPA buffer, and the lysates were probed with antibodies against p-ERK (Thr202/Tyr204), ERK, p-JNK (Thr183/Tyr185), JNK, p-p38 (Thr180/Tyr182), and p38. (B) C6 cells were pretreated for 1 h with γ-secretase inhibitors (2.5 μM DAPT and 1 μM L-685,486) and then exposed to 25 μM Cd for 6 h. (C) Cells were transfected with nicastrin siRNA (siRNA NCT) and non-targeting control siRNA(siRNA NON) for 48 h and treated with 25 μM Cd for 6 h. Total cell extracts were analyzed by western blotting using antibodies against p-ERK (Thr202/Tyr204), ERK, p-JNK (Thr183/Tyr185), JNK, p-p38 (Thr180/Tyr182), p38, and nicastrin. β-actin was used as a loading control.

To examine which MAPK signaling pathway is involved in the γ-secretase-mediated signaling pathways by CSE or Cd in C6 astrocyte cells, astrocyte cells were treated with 1, 10, or 25 μM of CdCl_2_ for 6 h and the resultant activation of MAPKs was analyzed by western blotting by mapping the phosphorylation status of extracellular signal regulated kinase (ERK)1/2, c-Jun N-terminal kinase (JNK), and p38 MAPKs using phosphorylation-specific antibodies antibodies (Fig 2A). At 10 μM, Cd exposure increased the phosphorylation of p38 MAPK (Thr180/Tyr182) and JNK (Thr183/Tyr185), but not ERK1/2 (Thr202/Tyr204). Treatment with 25 μM Cd increased the phosphorylation of JNK (p-JNK) and p38 MAPK (p-p38) in addition to ERK1/2 (p-ERK), suggesting that Cd activates all the MAPKs in C6 astrocyte cells. Next, we investigated whether Cd-mediated MAPK activation is regulated by γ-secretase activity. The γ-secretase inhibitors, L-685,486 and DAPT, partially blocked the 25 μM Cd treatment-induced phosphorylation of p38 MAPK (Fig 2B). However, the phosphorylation of ERK or JNK induced by Cd was not affected by inhibition of γ-secretase (Fig 2B). These results were further confirmed by examining the effect of knockdown of γ-secretase activity on the phosphorylation of MAPKs. Transfection of cells with siRNA targeted against nicastrin reduced the levels of nicastrin protein (Fig 2C). Depletion of nicastrin in C6 cells with a consequent knockdown of γ-secretase activity did not inhibit the Cd-dependent phosphorylation of ERK or JNK (Fig 2C). However, the increased phosphorylation of p38 MAPK elicited by Cd exposure was attenuated by nicastrin knockdown in C6 cells (Fig 2C). These results suggest that Cd-induced γ-secretase activity is involved in enhanced phosphorylation of p38 MAPK.

### γ-secretase mediates Cd-induced CREB phosphorylation

**Fig 3:**
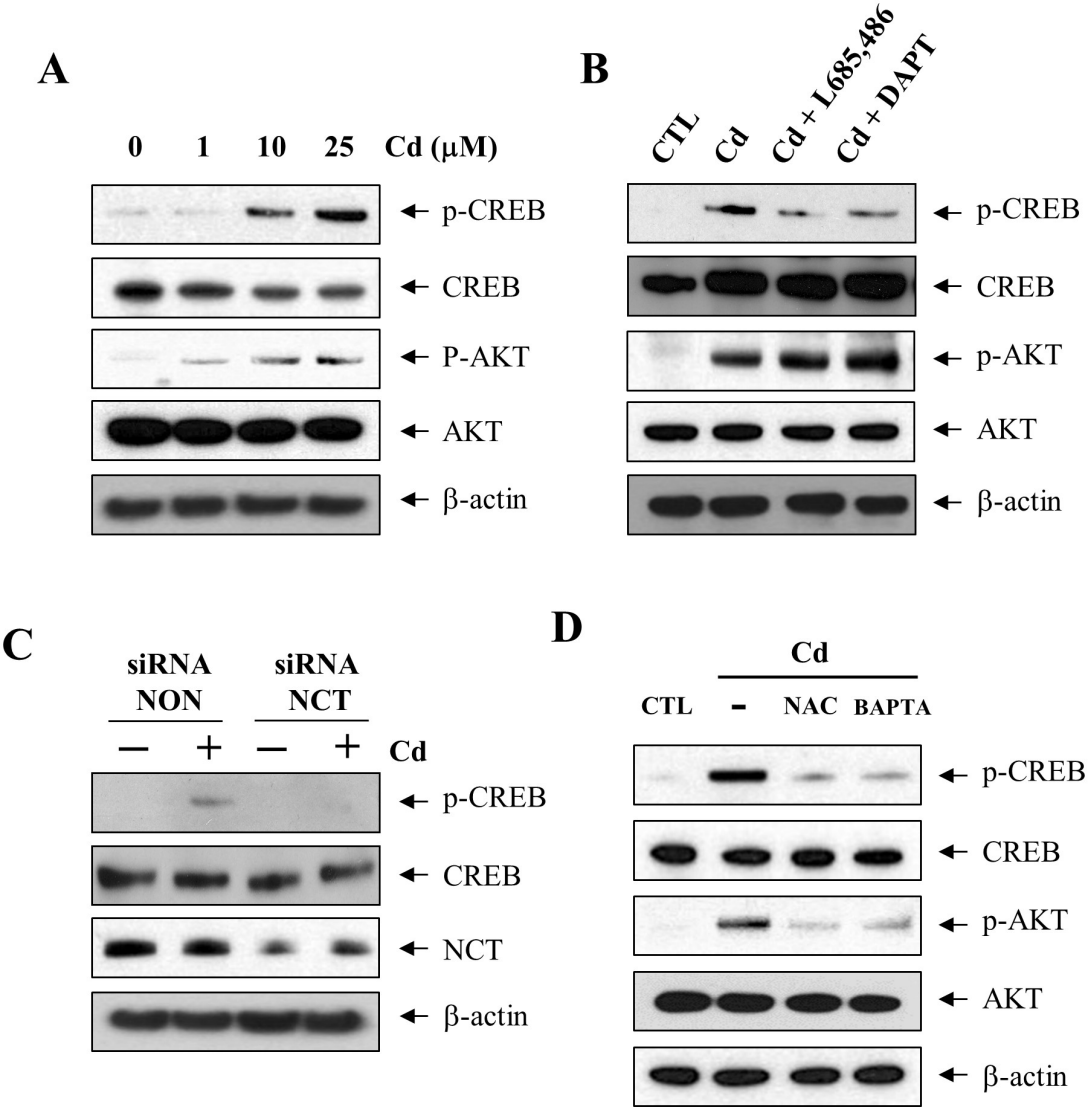
Phosphorylation of CREB by Cd is mediated by γ-secretase activation. (A) C6 cells were treated for 6 h with 0, 1, 10, and 25 μM Cd and lysed in RIPA buffer. (B) C6 cells were pretreated for 1 h with γ-secretase inhibitors (2.5 μM DAPT and 1 μM L-685,486) and then exposed to 25 μM Cd for 6 h. (C) Cells were transfected and incubated with siRNA against nicastrin (siRNA NCT) and non-targeting control (siRNA NON) for 48 h followed by treatment with 25 μM Cd for 6 h. (D) C6 cells were preincubated for 1 h in the presence of BAPTA-AM (10 μM) or NAC (10 mM), and then treated with 25 μM Cd. Total cell extracts were analyzed by western blotting using antibodies against p-CREB (Ser133), CREB, p-AKT (Thr308), and AKT. β-actin indicated equal loading of lysates.

Activated p38 MAPK can regulate the activation of transcription factors, such as ATF-2 and CREB [24]. Thus, we investigated the effect of Cd exposure on CREB phosphorylation with anti-phospho CREB antibody (Ser133). When C6 cells were treated with Cd (25 μM), CREB phosphorylation (p-CREB) was observed at 6 h (Fig 3A). To determine if γ-secretase is involved in the phosphorylation of CREB, C6 cells were pretreated with two γ-secretase inhibitors, DAPT and L-685,486. As expected, DAPT and L-685,486 treatment reduced the Cd exposure-dependent increased phosphorylation of CREB (Fig 3B). Furthermore, the knockdown of nicastrin by siRNA transfection also reduced the phosphorylation of CREB (Fig 3C). Considering that the AKT activated in the PI3K signaling pathway is a crucial upstream signal for CREB activation [25], we further assessed whether the AKT signaling pathway regulates the γ-secretase-dependent activation of CREB with AKT and phosphorylation-specific AKT antibodies (Thr308). The exposure of C6 cells with Cd induced the phosphorylation of AKT (p-AKT) in a dose-dependent manner (Fig 3A). However, pretreatment of cells with DAPT or L-685,486 could not block the Cd-induced AKT phosphorylation (Fig 3B). Together, these results suggest that AKT activation by Cd might be not involved in the γ-secretase-mediated CREB phosphorylation. We also examined whether Cd exposure-associated increase in ROS and Ca^2+^ activates the phosphorylation of CREB. To block the ROS or Ca^2+^ production, C6 cells were treated with antioxidant NAC or Ca^2+^ chelator BAPTA-AM, respectively. Both the drugs (10 mM NAC and 10 μM BAPTA-AM) decreased the phosphorylation of CREB induced by Cd (Fig 3D). These results suggest that Cd increases the intracellular Ca^2+^ or ROS, which induces γ-secretase–dependent phosphorylation of CREB through the activation of the p38 MAPK signaling pathway in C6 astrocyte cells.

### CSE and Cd induces the COX-2 expression via γ-secretase-mediated activation of p38 MAPK

**Fig 4:**
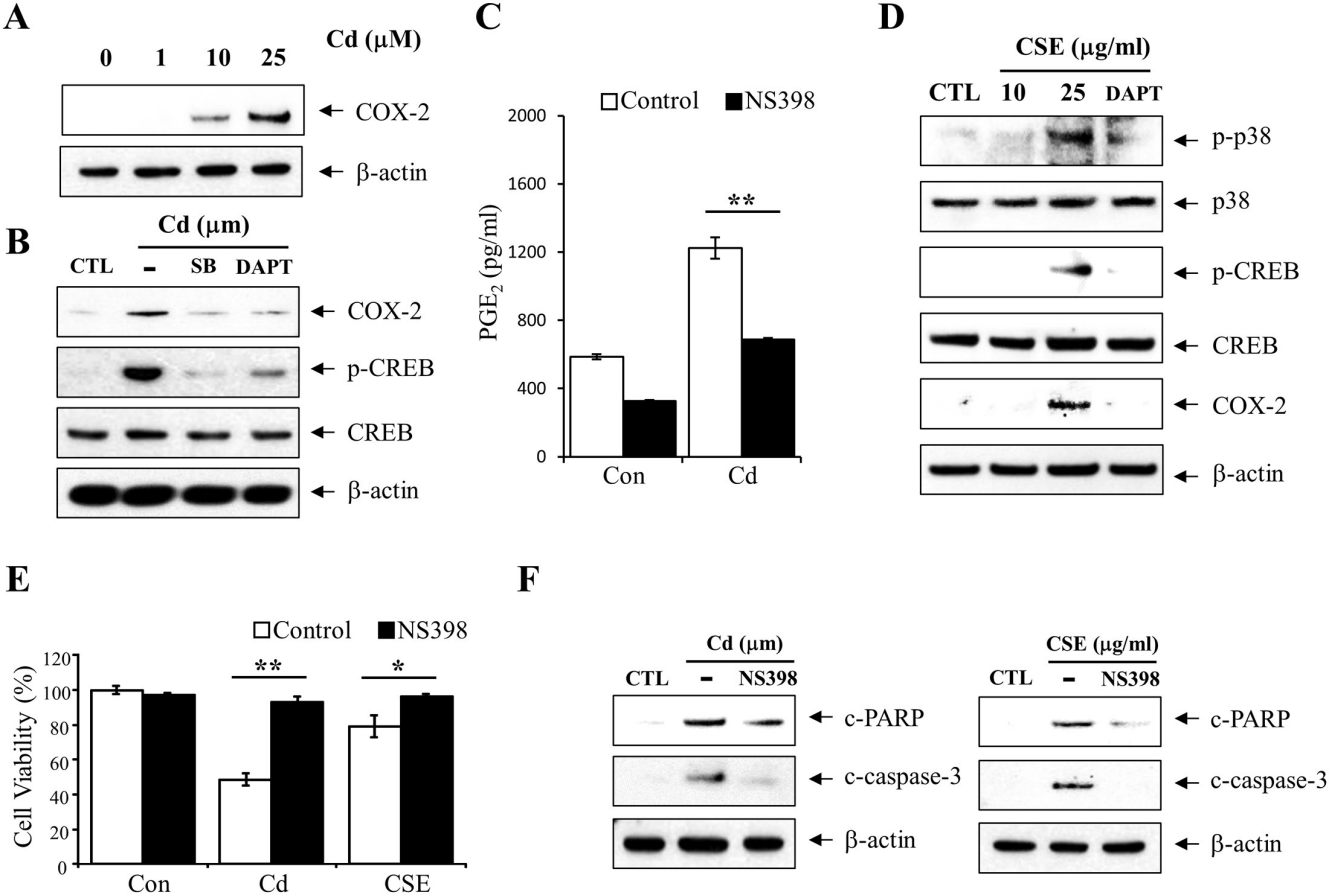
Cd and CSE increased COX-2 expression via γ-secretase activation. (A) C6 cells were treated for 6 h with 0, 1, 10, and 25 μM Cd, lysed in RIPA buffer, and the lysates were analyzed for COX-2 expression by western blotting using COX-2 antibody. (B) C6 cells were preincubated for 1 h in the presence of SB202190 (5 μM) or DAPT (2.5 μM). Total cell extracts were analyzed by western blotting using antibodies against COX-2, p-CREB (Ser133) and CREB. (C) C6 cells were treated for 6 h with 10 μM Cd with 1 hour pretreatment of COX-2 inhibitor (20 μM NS398). After Cd treatment, cells were washed 3 times with PBS and incubated for 24 hours. Cell supernatants were collected and measured secreted PGE_2_ level after incubation. (D) C6 cells were pretreated with 2.5 μM DAPT for 1 h and then incubated with 25 μg/ml CSE for 6 h. Total cell extracts were prepared and subjected to western blotting analysis using anti-p-p38 (Thr180/Tyr182), anti-p38, anti-p-CREB (Ser133), anti-CREB, and anti-COX-2 antibodies. β-actin was used as a loading control. (E) Effects of CSE or Cd-induced COX-2 overexpression on cell viability were evaluated by MTT assay. Cells were pretreated for 1 h in the presence or absence of COX-2 inhibitor (20 μM NS398) and then exposed to 25 μM Cd or 25 μg/ml CSE for 6 h. (F) Immunoblot analysis of apoptosis-related proteins. Cells were pretreated for 1 h in the presence or absence of 20 μM NS398 and then exposed to 25 μM Cd or 25 μg/ml CSE for 6 h. The lysates were probed with antibodies against c-caspase-3 (Asp175) and c-PARP (Asp214/215). β-actin was used as a loading control for lysates. Data are shown as the mean ± SD (n = 3), *P<0.05, **P<0.01, compared to control.

When C6 cells were treated with Cd for 6 h, protein levels of COX-2 were upregulated (Fig 4A). As we expected, Cd-induced COX-2 protein expression was not observed in the presence of γ-secretase inhibitor, DAPT (Fig 4B). Furthermore, pretreatment of cells with p38 MAPK inhibitor, SB202190 partially reduced the protein level of COX-2 and phosphorylation of CREB (Fig 4B). To confirm Cd induced COX-2 expression affects Prostaglandin E_2_ (PGE_2_) synthesis, we measured the secreted PGE_2_ levels in presence or absence of COX-2 inhibitor, NS-398 (Fig 4C). The result indicate that increased PGE_2_ was attributable to increased COX-2 that induced by Cd. These results suggest that γ-secretase-mediated p38 MAPK activation play an important role in inducing the expression of COX-2 protein. A moderate increase in p38 MAPK/CREB phosphorylation and COX-2 expression was detected in the cells treated with CSE (Fig 4D), which was reversed by the inhibition of γ-secretase activity Furthermore, CSE and Cd induced cytotoxicity in C6 cells, which was blocked by treatment with COX-2 inhibitor, NS-398 (Fig 4E). We also investigated whether COX-2 involved that Cd and CSE induced apoptosis (Fig 4F). Our data implicate that COX-2 expression by CSE and Cd regulates astrocytes apoptosis.

### NICD is involved in CSE/Cd-mediated increase in CREB phosphorylation and COX-2 expression

**Fig 5:**
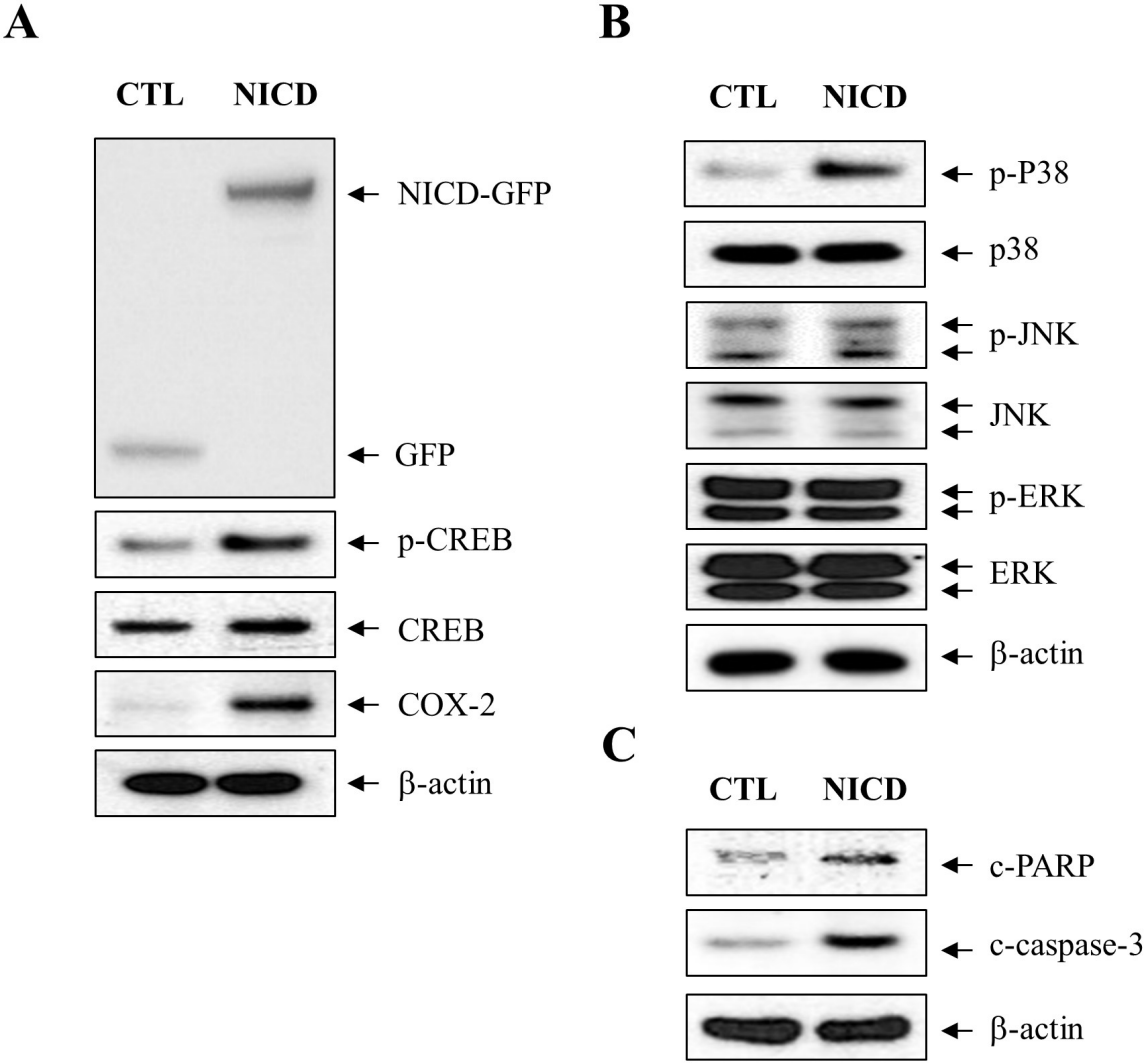
NICD is involved CSE/Cd-mediated CREB phosphorylation and induction of COX-2 expression. (A) C6 cells were transiently transfected with either the control GFP (CTL) or GFP-tagged NICD (NICD). After transfection, cells were lysed in RIPA buffer and the total cell extracts were subjected to western blotting using antibodies against GFP, p-CREB (Ser133), CREB, and COX-2. (B) Cell extracts were analyzed by immunoblotting with antibodies against p-p38 (Thr183/Tyr185), p38, p-ERK (Thr202/Tyr204), ERK, p-JNK (Thr183/Tyr185), and JNK. (C) Cell extracts were subjected to western blotting analysis using c-PARP (Asp214/215) and c-caspase-3 (Asp175) antibodies. β-actin was used as a loading control.

We next investigated whether increase in NICD is involved in p38 MAPK/CREB signaling and COX-2 expression. C6 cells were transiently transfected to express either control GFP or GFP-NICD from IRES-eGFP vector. After 48 h of incubation, phosphorylation of p38 MAPK and CREB, and COX-2 levels were increased by overexpression of NICD (Fig 5A and 5B). However, the phosphorylation of ERK or JNK was not affected by NICD transfection (Fig 5B). These results suggest that phosphorylation of p38 MAPK is involved in the NICD-induced COX-2 expression in C6 cells. Furthermore, we investigated whether overexpressed NICD affects astrocyte cell death. The cleavage of caspase-3 and PARP was increased in NICD transfected cells compared to the control cells (Fig 5C). These results suggest that NICD production is involved in CSE/Cd exposure-mediated astrocyte death.

## Discussion

**Fig 6:**
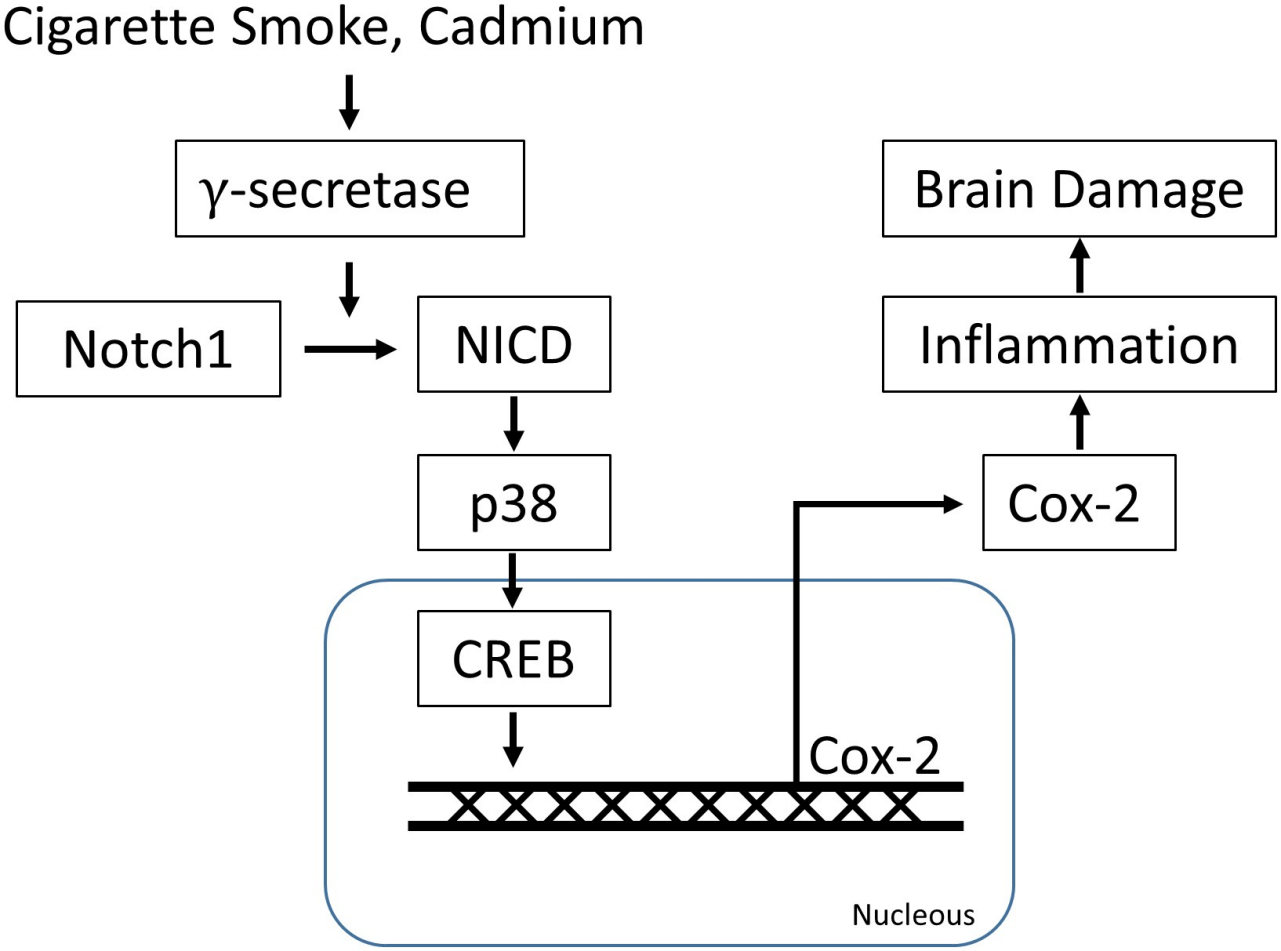
Schematic diagram summarizing that CSE/Cd induced COX-2 expression through γ-secretase-mediated p38 MAPK activation. Exposure of Cd and CSE result in activation of γ-secretase that regulates Notch1 cleavage and release of NICD in astrocyte. Released NICD enhances p38 phosphorylation that activates CREB signaling. By phosphorylation of CREB, the expression of COX-2 is stimulated which eventually leads to apoptosis.

The present study reveals a mechanism of COX-2 upregulation and highlights the role of p38 MAPK in mediating the hazards associated with exposure to cigarette smoke or its carcinogen, Cd. We found that CSE and Cd increased γ-secretase-mediated NICD production in C6 astrocytes cells. CSE and Cd-induced phosphorylation of CREB and p38 MAPK was specifically suppressed by the inhibition of γ-secretase. NICD production resulted in an increase in COX-2 expression levels and phosphorylation of p38 MAPK and CREB. Furthermore, Cd or CSE induced cytotoxicity was blocked by γ-secretase inhibitor DAPT or COX-2 inhibitor NS-398. Thus, the current study suggests that CSE and Cd modulate γ-secretase-mediated Notch signaling, resulting in the initiation of p38 MAPK/CREB signaling cascades to induce COX-2 expression and apoptosis in C6 astrocytes cells (Fig 6).

We have shown previously that Cd induces COX-2 expression in part by the activation of presenilin1/γ-secretase (16). However, the underlying mechanism was unknown. In this study, we demonstrate that presenilin1/γ-secretase is involved in the induction of COX-2 expression upon exposure to cigarette smoke. Furthermore, we first showed that γ-secretase-mediated NICD production and p38 MAPK/CREB signaling pathways are involved in the COX-2 expression by cigarette smoke. Cd exposure leads to the activation of various signaling proteins, including ERK, JNK, p38, protein kinase C, and phoshatidylinositol 3-kinase (PI3K)/AKT [26–28]. JNK and p38 MAPK signaling cascades induced by harmful stimuli promote neuronal cell death [24]. MAPK pathways contribute to ischemic stroke by increasing the pro-inflammatory cytokines production [29]. Recent report suggests that CSE induces COX-2 over-expression in macrophages through ERK and p38 MAPK signaling, but not JNK [30]. γ-secretase inhibitors reduce the ischemia-induced increase in phosphorylation of JNK/c-Jun in neuronal cells [31]. However, a previous report showed that PS2, as an upstream regulator, participates in the intracellular p38 MAPK signaling cascade [27]. Our findings in the present study provide substantial evidence showing the role of γ-secretase as an upstream regulator of p38 MAPK activation in CSE-exposed C6 cells. Increased p38 MAPK activation after ischemic stroke has been observed in astrocytes and microglia [32]. These observations lead us to examine the involvement of γ-secretase in p38 MAPK phosphorylation activated by CSE and Cd. The data revealed the inhibitory effect of γ-secretase inhibitors or nicastrin depletion by siRNA on CSE and Cd-induced p38 MAPK phosphorylation, suggesting that γ-secretase modulates the p38 MAPK signaling pathway upon exposure to cigarette smoke and Cd.

Smoking is a strong risk factor for a variety of diseases, including cerebrovascular disorders and thus, remains a major cause of death worldwide [33]. Cigarette smoke is one of major source of Cd exposure. In epidemiologic studies, it has been shown that environmental cadmium exposure was associated with increased risk of stroke [7, 34]. Cd induces phosphorylation of CREB [35], which is also observed in the blood of smokers [36]. Cigarette smoke extracts induces the activation of protein kinase A and CREB signaling pathway [16]. Enhanced p38 MAPK phosphorylation can activate its substrates including ATF-2 and CREB. In H9c2 rat heart cells, however, Cd exposure reduced the phosphorylation of AKT [37]. According to our findings, Cd-induced phosphorylation of AKT was not blocked by the treatment with γ-secretase inhibitors (Fig 3); thus, γ-secretase might not mediate the PI3K/AKT signaling cascades activation induced by Cd. In this report, we have shown for the first time that γ-secretase activation induces the phosphorylation of CREB in the CSE-exposed C6 cells. Furthermore, CREB phosphorylation by Cd was shown to be mediated by Ca^2+^ and reactive oxidative stress (ROS) (Fig 3D). Although the precise role of the signaling pathway triggered by Cd-induced γ-secretase mediated phosphorylation of CREB remains to be determined, our results highlight the pathway of ROS and Ca^2+^ in the process of γ-secretase-mediated CREB phosphorylation by Cd.

Since astrocytes contribute to neuroprotection, dysfunction of astrocytes could induce neuronal damage. There is increasing evidence of astrocyte apoptosis during brain injury [38]. Dysfunction of astrocytes induces the progress of stroke [39]. Our study also showed that CSE and Cd treatment resulted in significant increase in apoptosis of C6 astrocyte cells, which was blocked by inhibition of γ-secretase or COX-2. Furthermore, Cd exposure in C6 astrocyte cells increased PGE_2_ levels, one of major downtstream product of COX-2 (Fig 4). Since PGE_2_ has been regarded as a major mediator of inflammation and caspase-dependent apoptosis [40, 41], PGE_2_ have considered that regulates COX-2 induced neurotoxicity [42].

Given that the treatment of COX-2 inhibitors exhibits significant neuroprotective effect under exposure of Cd or CSE (Fig 4), increased PGE_2_ levels after Cd exposure might be involved in the Cd-mediated apoptosis. Astrocytes can regulate extracellular glutamate primarily through the astrocytic glutamate transporter-1 and the Na(+)-dependent glutamate/aspartate transporter (GLAST) [43]. Cd reduced GLAST expression through the activation of CREB, suggesting that CREB phosphorylation by Cd in astrocytes might reduce the astrocytic glutamate uptake function and thus, may induce damage after CNS injury [44].

In conclusion, we provide evidence for the γ-secretase-mediated p38 MAPK/CREB signaling in C6 astrocyte cells by Cd and cigarette smoke. Our results suggest that γ-secretase-mediated NICD production by cigarette smoke and Cd exposure may be an important step for COX-2 expression via p38 MAPK-mediated signaling cascade.

## Abbreviations

Cd: Cadmium
COX-2: Cyclooxygenase-2
CREB: Cyclic AMP response element-binding protein
CSE: cigarette smoke extracts antioxidant
NICD: Notch1 intracellular domain
